# Modeling the Potential Climatic Suitability and Expansion Risk of *Tuta absoluta* (Meyrick, 1917) (Lepidoptera: Gelechiidae) Under Future Climate Scenarios

**DOI:** 10.3390/insects16020185

**Published:** 2025-02-09

**Authors:** Tai Gao, Rui Feng, Zibo Liu, Zengrong Zhu

**Affiliations:** 1 College of Agriculture and Biotechnology, Zhejiang University, Hangzhou 310058, China; gaotai36@outlook.com (T.G.); 22216247@zju.edu.cn (R.F.); 2 Academy of Forestry Inventory and Planning, National Forestry and Grassland Administration of China, Beijing 100714, China; liuzibo@gjlcjghy.wecom.work

**Keywords:** tomato leaf miner, invasive pests, climate change, pest management

## Abstract

In this study, we predicted the global climatic suitability of *Tuta absoluta*, using maximum entropy (MaxEnt) modeling. We used species’ natural occurrence records in 1981–2024 and environmental variables in 1981–2010, reflecting near-current climate conditions, for modeling. The occurrence records used for modeling excluded the data from greenhouses and summer-only presence. The optimized MaxEnt models demonstrated an excellent predictive performance; Jaccard’s and Sørensen’s indices were greater than 0.8. Temperature, particularly the mean daily air temperature in February (tas2), was identified as the primary influencing factor. Projections based on five global climate models (GCMs) and four shared socioeconomic pathways (SSPs) indicated an increasing risk of *T. absoluta* expansion. Under SSP126, the lowest-risk period (2011–2040) exhibited a 7.08% increase in suitable areas, while SSP370 during the highest-risk period (2071–2100) projected an 18.13% increase relative to near-current conditions. Model outputs underestimated the pest’s actual distribution, underscoring its invasive potential. We recommend stringent quarantine measures, particularly for artificial facilities that support overwintering, to mitigate future invasions. These findings provide critical insights for policymakers and agricultural stakeholders to safeguard global tomato production against this invasive threat.

## Simple Summary

In this study, we explored the current and future global suitability of *Tuta absoluta*, a highly invasive pest originating from South America that threatens tomato production worldwide. By analyzing the environmental data and pest occurrence records, we identified areas where the climate is suitable for this pest. Our findings reveal that climate change may significantly expand the regions where *T. absoluta* can thrive. Known for its ability to spread rapidly and adapt to various environments, this pest poses a serious risk to agricultural systems. To limit its spread, governments and agricultural stakeholders should consider stricter quarantine measures, particularly in areas currently unsuitable for the pest. These measures could help protect greenhouses and tomato-growing regions from potential infestations. Our research highlights the need for proactive strategies to safeguard global food security and reduce the economic impacts of invasive pests.

## 1. Introduction

The tomato leaf miner, *Tuta absoluta* (Meyrick, 1917) (Lepidoptera: Gelechiidae), is native to South America but has invaded all major continents except Oceania and Antarctica; it has thus become one of the most economically significant pests of *Solanum lycopersicum* L. [1]. Beyond tomatoes, this pest threatens other solanaceous crops, such as *S. tuberosum* L., *S. melongena* L., and *Capsicum annuum* L., raising concerns about its broader agricultural impact [2]. The larval stage of *T. absoluta* is particularly destructive, as larvae feed on the mesophyll tissue of leaves, burrow into stems, and damage fruits, leading to necrotic lesions, leaf mining, and fruit deformation [3]. This feeding activity not only reduces photosynthetic capacity but also facilitates secondary infections by pathogens [3]. In contrast, adult moths do not directly harm plants but play a critical role in reproduction and the spread of the pest [4]. These challenges underline the need for effective monitoring and management strategies to mitigate its spread and economic consequences.

*Tuta absoluta* was first documented in Peru in 1917 [5]. By the 1990s, it had spread to nearly all South American countries. It was first reported outside of South America in eastern Spain in 2006 and it has since spread to all European countries, especially those in the Mediterranean region [1,6]. In 2008, *T. absoluta* was first reported in Morocco and has subsequently spread to Egypt and several other African countries [7]. From 2012 to 2017, *T. absoluta* extended its range to Southern Africa and Central Asia [8]. In eastern Europe, Western and Central Asia, and Northern and Southern Africa, it has been reported to spread at a rate of 800 km per year [9].

*Tuta absoluta* was detected in the northwestern Xinjiang Province, China, a region previously predicted unsuitable for *T. absoluta* by several ecological niche models (ENMs) [10,11,12,13,14,15]. *Tuta absoluta* exhibits high resistance to cold and heat and can survive for several days at 0 °C [16]. However, the 90% lethal temperature of *T. absoluta* indicated that it may struggle to overwinter in most northern and central regions of Xinjiang [17]. The lack of predictive accuracy in previous ENMs may be attributed to the rapid spread of *T. absoluta* and the absence of critical information on its environmental niche during modeling [12,13,18,19]. Furthermore, with global climate change, previously unsuitable habitats may potentially become habitable [10,14,20,21,22]. In China, *T. absoluta* has caused yield losses exceeding 80% in severely infested regions, resulting in substantial economic damage [23]. Given the uncertainties associated with the impact of future climate change on the predicted environmental suitability of *T. absoluta*, along with its wide occurrence records, clarifying the effects of various environmental factors on the environmental suitability of *T. absoluta* could aid in developing strategies to control the spread of this pest [24].

The maximum entropy (MaxEnt) model, widely used in predicting species distribution, harnesses occurrence records and environmental variables [25]. It provides several advantages for predicting the environmental suitability of invasive species. Specifically, the MaxEnt model is adept at handling presence-only data, a common scenario in invasive species research [25]. Furthermore, it effectively integrates nonlinear and interactive environmental variables, thus enhancing the accuracy of suitability predictions [26]. MaxEnt model also demonstrates a robust performance with limited occurrence records, making it particularly suitable for invasive species studies [27]. The MaxEnt model has been extensively applied in various studies focusing on predicting environmental suitability for numerous invasive alien species. This includes recent significant invasive insect events, such as *Spodoptera frugiperda* (J.E. Smith) (Lepidoptera: Noctuidae) and *Hylurgus ligniperda* (Fabricius) (Coleoptera: Curculionidae), invading China [28,29]. Over short historical periods, species’ ecological niches are often conserved, with a low rate of niche evolution [30]. However, when an invasive species has not yet reached ecological equilibrium with its new habitat, there is a risk of underestimating its environmental niche, which could lead to an underestimation of its potential geographic range of invasion [31]. Accurately predicting the environmental suitability of invasive species is particularly challenging when key ecological niche data are unavailable, as this can introduce considerable uncertainty into modeling efforts. When ENMs attempted to predict potential distributions solely based on the occurrence data from either native or invaded regions, the model outputs yielded significantly different results [32].

In this study, our primary objective was to clarify the potential impact of climate change on the distribution of *T. absoluta* by analyzing changes in the species’ natural occurrence points during its global spread. Additionally, we aimed to model the environmental suitability of *T. absoluta* under near-current and future climate scenarios, using its natural occurrence records and incorporating more recent invasion data. Furthermore, we will investigate the climatic and anthropogenic factors driving the rapid expansion of *T. absoluta* relative to other invasive alien species. This novel approach accounts for the species’ response to climate change and its ongoing global spread, which is expected to improve the accuracy of invasion and spread risk assessments for invasive species.

## 2. Materials and Methods

### 2.1. Environmental Variables

The availability of high-resolution climatic data has greatly aided studies employing MaxEnt modeling [33]. The latest version of the Climatologies at High resolution for the Earth’s Land Surface Areas (CHELSA, available online: https://chelsa-climate.org/, accessed on 1 June 2024) is v2.1. The accuracy of the temperature data in the CHELSA climatological dataset is similar to that of other datasets; however, the accuracy of the precipitation data in the CHELSA climatological dataset is superior to that of other datasets [33]. The CHELSA data were available for 1981–2010 under near-current climate conditions. We resampled the CHELSA climatological raster data from a resolution of 30 arc-sec to 2.5 arc-min using the ArcGIS Desktop software v10.8.1 (available online: https://www.esri.com/, accessed on 15 February 2022, paid).

We selected environmental variables for the MaxEnt modeling process from a pool of 67 climatic variables provided by CHELSA, in addition to elevation data (Table S1) [22,33]. To examine the relationships between the environmental variables, we used SPSS software v20.0.0 (available online: https://www.ibm.com/spss, accessed on 15 February 2022, paid) to conduct Pearson correlation coefficient analysis [34]. The Variance Inflation Factor (VIF) was calculated using R software v3.6.0 (available online: https://www.r-project.org/, accessed on 26 February 2022, free) to quantify the extent to which standard errors were inflated due to the multicollinearity of environmental variables [35]. When the absolute value of the Pearson correlation coefficient exceeded 0.8 and VIF values exceeded 10, it indicated significant collinearity or redundancy among the environmental variables [36]. During the environmental variable selection process, we retained the environmental variable that made a higher percentage contribution to the MaxEnt model and excluded those with lower contributions, based on the output (*.html file, under the “Analysis of variable contributions” section) generated by the Maxent software v3.4.4 (available online: https://biodiversityinformatics.amnh.org/open_source/maxent/, accessed on 2 August 2021, free).

The Coupled Model Intercomparison Project Phase 6 (CMIP6) data, derived from the CHELSA dataset and shared socio-economic pathways (SSPs), included SSP126 (represents a scenario characterized by low greenhouse gas emissions, with CO_2_ emissions targeted to reach net zero around 2075), SSP370 (corresponds to a scenario of high greenhouse gas emissions, where CO_2_ emissions are projected to double by 2100), and SSP585 (indicates a scenario with very high greenhouse gas emissions, with CO_2_ emissions expected to triple by 2075). SSPs can provide insights into the correlations between socio-economic development and climate scenarios [37]. CMIP data are essential for examining past climate changes and predicting of future climate changes. Periods for which CMIP6 data from the CHELSA dataset were available included 2011–2040, 2041–2070, and 2071–2100. We preselected CMIP6 global climate models (GCMs), including UKESM1-0-LL, IPSL-CM6A-LR, MRI-ESM2-0, MPI-ESM1-2-HR, and GFDL-ESM4 shown in Table 1 [33]. The priority of GCMs follows the suggestions of the phase 3b of the Inter-Sectoral Impact Model Intercomparison Project (ISIMIP3b) protocol [38]. Concerning climate sensitivity, the selection of these five GCMs effectively captured the breadth of the CMIP6 ensemble. This subset encompasses three GCMs demonstrating low climate sensitivity (MRI-ESM2-0, MPI-ESM1-2-HR, and GFDL-ESM4) and two GCMs displaying high climate sensitivity (UKESM1-0-LL and IPSL-CM6A-LR), thus providing a comprehensive representation [38,39]. The five GCMs under consideration exhibit varying climate sensitivities, ranked by their equilibrium climate sensitivity (ECS) values, as reported by CarbonBrief (available online: https://www.carbonbrief.org/cmip6-the-next-generation-of-climate-models-explained/, accessed on 1 June 2024) (Table 1). To eliminate individual biases that may arise from using a single GCM, this study combined the predictions from the five GCMs into a multi-GCM result to reflect the species’ climatic suitability under future climate conditions.

### 2.2. Occurrence Records

We collected occurrence records for *T. absoluta* from the following sources: (1) Specimen records deposited in the Institute of Insect Sciences (Zhejiang University, Hangzhou, China); (2) Field survey data collected in China in 2021–2024; (3) Published studies [21,40,41,42,43,44,45,46,47,48]; and (4) Global biodiversity databases, including the Global Biodiversity Information Facility (GBIF, available online: https://www.gbif.org/, accessed on 8 October 2024), Center for Agriculture and Bioscience International (CABI, available online: https://www.cabi.org/, accessed on 8 October 2024), and European and Mediterranean Plant Protection Organization (EPPO, available online: https://www.eppo.int/, accessed on 8 October 2024).

We categorized the collected occurrence records into three datasets. (1) The basic dataset: This dataset represents a fixed-niche space and includes species’ natural occurrence records in 1981–2010, corresponding to the period of CHELSA environmental variables. To ensure a natural representation of the species distribution, records from greenhouses and those restricted to the summer season were excluded. (2) The expanded dataset: Representing an expanding-niche space, this dataset assumed a limited response to climate change in 2011–2024 as *T. absoluta* continued its global spread. During this period, the species’ habitats did not shift significantly toward higher latitudes or altitudes. The expanded dataset extended the species’ natural occurrence records to include the period from 2011 to 2024, while maintaining the exclusion of greenhouse and summer-only records. (3) The excluded dataset: This dataset retained records from greenhouses and summer-only occurrences separately for further analysis of non-climatic factors, specifically the role of anthropogenic drivers in the species’ rapid expansion.

We used the ENMTools package in R software to eliminate spatial correlations among the species’ natural occurrence records used for modeling, thereby preventing model overfitting [49]. In cases where multiple occurrence points were located within a single cell (or pixels, where each cell contains a value representing information like temperature), the ENMTools package v1.1.2 retained only one point to ensure that information from each cell with an occurrence was captured while avoiding redundancy. We used GS+ software v9.0 (available online: https://www.geoengineer.org/software/gs, accessed on 15 February 2022, paid) to calculate Moran’s Index (Moran’s *I*) for the species’ natural occurrence records, which quantifies spatial correlation on a scale ranging from −1.0 to 1.0, depending on the extent and direction of correlation [50]. By removing records with a high spatial correlation, we adjusted the Moran’s *I* of the dataset used for modeling to below 0.2 [51].

### 2.3. MaxEnt Model Optimization

Given the widespread invasion of *T. absoluta* across diverse global regions, its distribution dynamics has continuously evolved [9]. Discerning true absence has become a challenging endeavor. Furthermore, an increase in pseudo-absences might have downplayed the natural drivers of species occupancy, thereby reducing the predicted environmental suitability within the native range [28]. Consequently, we chose to employ presence-only algorithms.

We performed the following methods within the Maxent software: generated response curves between the important environmental variables and the probability of presence; conducted a jackknife analysis to assess variable importance; utilized the cloglog output mode; and set the output file format to *.asc format [52,53,54,55]. To predict *T. absoluta*’s environmental suitability under future climate conditions, we ensured that the filenames of the future climate variables matched those of the near-current climate variables. Subsequently, these files were loaded into the “Projection layers directory/file”.

In this analysis, we employed the following parameters: a random seed, which operates similarly to bootstrapping, where each run uses a different random seed, and a random test percentage set at 25% [25]. Given that *T. absoluta* may still be in a phase of rapid expansion, we used the “background.buffer” function in the ENMTools package in R software to select pseudo-absence points within a buffer zone surrounding the species’ natural occurrence records [9,56,57]. We used the kuenm package in R software to calculate the regularization multiplier (RM) and feature classes (FCs) [58]. The RM values ranged from 0 to 5, with intervals of 0.1. The FCs included linear (L), quadratic (Q), hinge (H), product (P), and threshold (T). We computed the corrected Akaike Information Criterion (AICc) values under different parameters using the R package kuenm [58]. Model complexity was assessed using the AICc value, and we selected the optimal RM and FC based on the model with the lowest AICc value [59]. We tested a total of 31 different FC combinations, encompassing independent and combined cases of L, Q, H, P, and T [60]. To reduce the randomness of the predicted results, the model was run 20 times under the same settings, and the final outcome was presented as the average of these replicates [61].

### 2.4. Classification and Variability of Areas with Environmental Suitability

The predicted environmental suitability of *T. absoluta* was analyzed using ArcGIS Desktop software. Model outputs were transformed into consensus maps using the threshold that maximized the true skill statistics (max-TSS) [62]. In cases where absence data were unavailable, utilizing of the max-TSS remained an appropriate approach, even when employing randomly selected points as surrogate absence data [63]. To compute the max-TSS, we employed R software to randomly generate absence points until the prevalence rate reached 50% [64,65]. Subsequently, we utilized the “Reclassify” function in ArcGIS Desktop software to calculate the area of suitable and unsuitable habitats.

In addition to analyzing the average predicted outputs from 20 replicates, we aimed to evaluate the variability in environmental suitability by calculating the standard deviation across all model outputs. Furthermore, we calculated the percentage of the total area where the model achieved unanimous 95% agreement, classified as consistent areas, and classified the rest as inconsistent areas.

In future climate scenarios, using various GCMs with the MaxEnt models could yield different predictions of environmental suitability. To elucidate the variability arising from this array of GCMs, we calculated the mean environmental suitability across distinct periods and climate scenarios using five GCMs.

### 2.5. Model Performance and Influence of Environmental Variables

To assess model overfitting, we employed several criteria: the minimum training presence omission rate (OR_mtp_) and the 10th percentile training presence omission rates (OR_10_) [27]. The model’s predictive performance was considered excellent when both OR_mtp_ approached 0 and OR_10_ neared 0.1 [66].

We evaluated the predictive accuracy of the MaxEnt models by evaluating the area under the receiver operating characteristic (ROC) curve (AUC) and the AUC value of the partial-area ROC (P-ROC AUC) [67,68]. The AUC values ranged from 0.5 to 1. An AUC in the range of 0.5 to 0.6 indicated a negligible predictive capability, 0.6 to 0.7 suggested limited predictive prowess, 0.7 to 0.8 implied a moderate predictive capacity, 0.8 to 0.9 indicated substantial predictive competence, and 0.9 to 1.0 signified a strong predictive capacity [67]. Regarding the P-ROC AUC, we applied a 5% error rate (E = 0.05) to calculate the AUC ratios, specifically AUC ratio = AUC_E_/AUC_0.5_, using Niche Analyst software v3.0 (available online: https://nichea.sourceforge.net/, accessed on 15 February 2022, free). Here, AUC_E_ represents the area under the curve within the specified error rate (E = 0.05), while AUC_0.5_ denotes the area under the curve for a random prediction (AUC = 0.5). The AUC ratio quantifies the improvement of the model’s predictive performance over a random prediction, with values greater than 1.0 indicating a better performance than random. This approach allows us to evaluate the model’s accuracy in the context of partial-area ROC analysis [68].

Furthermore, our study employed the true skill statistics (TSS) developed by Hanssen and Kuipers [69], the Kappa statistic introduced by Cohen [70], and the Boyce index proposed by Boyce et al. [71]. These metrics, namely TSS, Kappa, and Boyce index, operate within a range of −1 to 1. A value approaching 1 indicates a high level of accuracy, with predictions ranging from good to perfect. Values near 0 suggest predictions similar to random chance, while values approaching −1 imply counter-predictions [72]. The procedure for generating random points followed the approach used for computing the max-TSS values [64].

Since the use of indices, such as TSS, may mislead model performance in the absence of high-quality presence-background data, we also considered Jaccard’s and Sørensen’s indices to assess the model’s accuracy [73,74]. Jaccard’s and Sørensen’s indices range from 0 to 1, with values approaching 1 indicating a high level of accuracy, reflecting predictions ranging from poor to perfect [73].

All model evaluation metrics, including the AUC, AUC ratio, TSS, Kappa, Boyce index, Jaccard’s index, and Sørensen’s index, were calculated exclusively using the independent test dataset to avoid overfitting and mitigate the risk of inflated accuracy estimates (i.e., the “accuracy trap”). This approach ensures a robust assessment of model generalizability and predictive performance on unseen data [75].

## 3. Results

### 3.1. Key Parameters and Quality Assessment of MaxEnt Models

**Figure 1:**
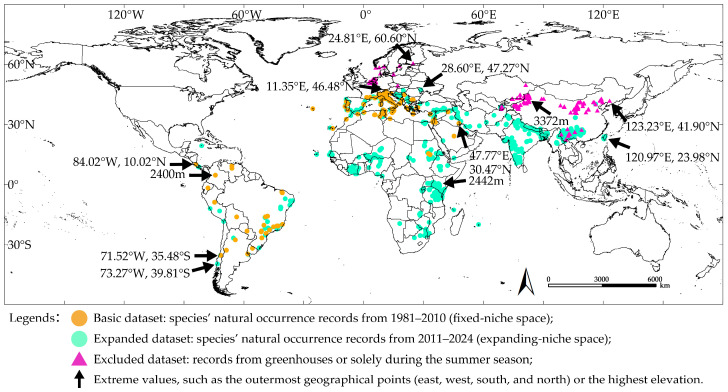
Natural occurrence records of *T. absoluta* in 1981–2024, and records from greenhouses and summer-only presence.

By comparing the distribution range of *T. absoluta*’s natural occurrence records in 1981–2010 and 2011–2024, we observed an increase in the population density within its established regions in South America and the Mediterranean, along with numerous new occurrence records (Figure 1). After 2010, *T. absoluta* expanded its range to invade regions in Central and Southern Africa, Central Asia, and East Asia (Figure 1). Specifically, in 2011–2024, the western boundary of the natural occurrence records remained consistent with the basic dataset, located at 84.02° W, 10.02° N in Central America. The southern boundary extended approximately 4.33° toward higher latitudes in Southern South America, while the northern boundary shifted approximately 0.79° toward higher latitudes in Europe. The eastern boundary showed the most significant expansion, extending approximately 73.20° from western to eastern Asia. The highest elevation increased by around 42 m (Figure 1).

Records from greenhouse and summer-only presence expanded further to the north and east, with their northern and eastern boundaries extending approximately 14.12° and 2.26° beyond the species’ natural occurrence records, respectively (Figure 1). The highest elevation recorded increased by approximately 930 m (Figure 1).

These results suggest that the natural occurrence records of *T. absoluta* are minimally influenced by climate change in 2011–2024. *Tuta absoluta* is currently in a phase of rapid expansion, but its habitat has not significantly shifted to higher latitudes or elevations (Figure 1). The CHELSA environmental variables for the period 1981–2010 can be used to predict the species’ environmental suitability under present climate conditions.

**Figure 2:**
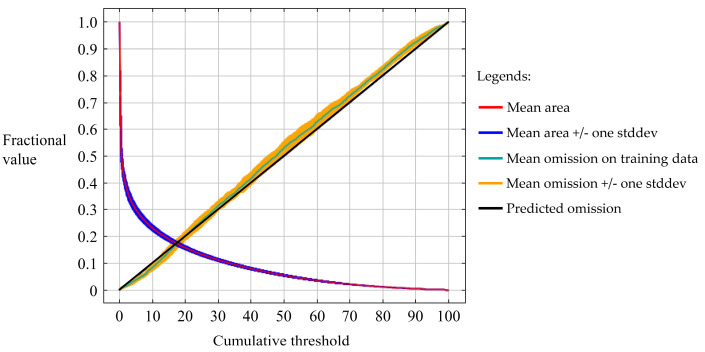
The mean omission curve of the MaxEnt model.

In the overfitting test of MaxEnt models, OR_mtp_ and OR_10_, performed well and approached the expected values (Table 2). Regarding the predictive accuracy of the MaxEnt models, the results show AUC values are greater than 0.9, AUC ratios are greater than 1, TSS is greater than 0.9, Kappa is greater than 0.8, the Boyce index is greater than 0.9, Jaccard’s index is greater than 0.8, and Sørensen’s index is greater than 0.9 (Table 2). The mean omission curve of the test data exhibits only a modest deviation from the predicted omission curve (Figure 2). Collectively, this indicated that the models do not suffer from overfitting and possess excellent predictive accuracy.

Under the condition that Pearson correlation coefficient was <0.8 and VIF was <10, and ranked by their percentage contribution, we selected seven climatic variables for the modeling process; the selected climatic variables included mean daily air temperature in February (tas2), precipitation seasonality (bio15), monthly precipitation in February (prec2), mean monthly precipitation amount of the coldest quarter (bio19), monthly precipitation in July (prec7), precipitation amount of the wettest month (bio13), and mean diurnal air temperature range (bio2) (Table 3).

**Figure 3:**
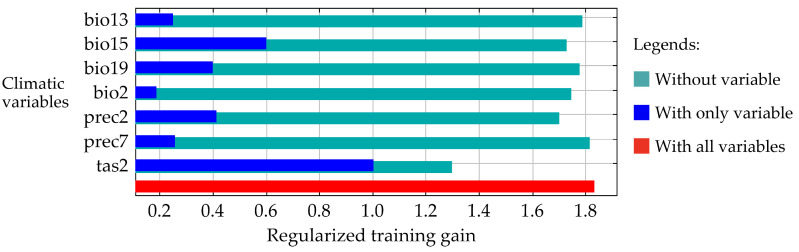
Results of jackknife analyses on climatic variables in the MaxEnt model.

The jackknife analysis of climatic variables indicated that tas2 had the most significant impact on the climatic suitability for *T. absoluta*. Additionally, this variable contained the most unique information not found in the other variables (Figure 3).

**Figure 4:**
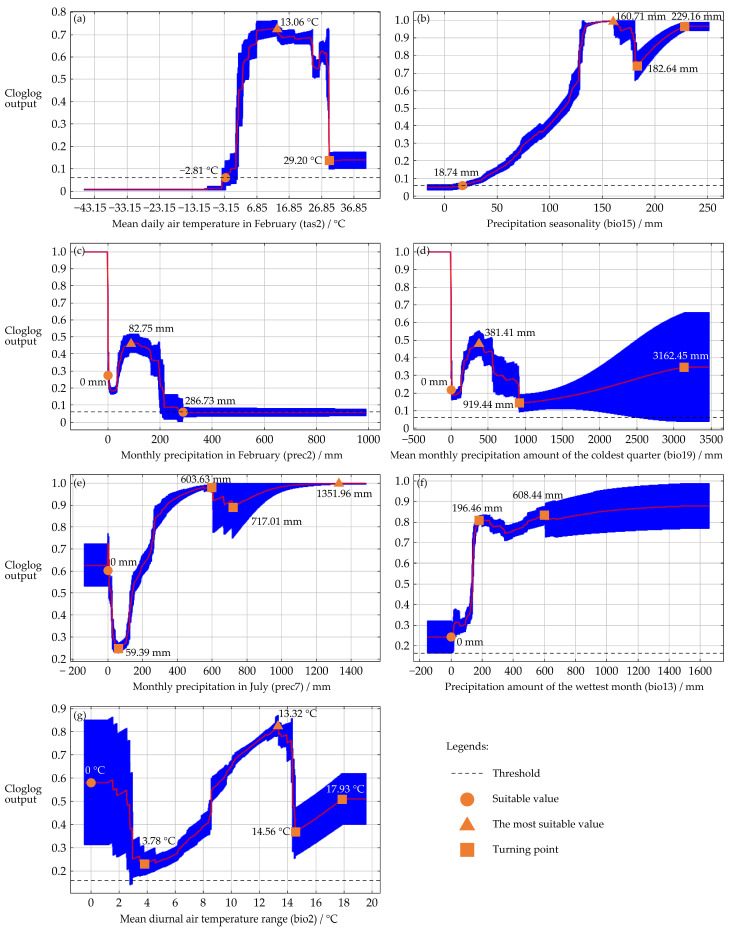
The response curves generated by the MaxEnt model demonstrate the connection between climate variables and the climatic suitability of *T. absoluta*: tas2 (**a**), bio15 (**b**), prec2 (**c**), bio19 (**d**), prec7 (**e**), bio13 (**f**), and bio2 (**g**). The curves represent the mean response of 20 replicate Maxent runs (red) and the mean ± one standard deviation (blue, with two shades for categorical variables).

The response curves illustrate the relationship between climatic variables and the predicted climatic suitability of *T. absoluta*. The probability of *T. absoluta*’s presence increased when tas2 was greater than −2.81 °C, reached an optimum at 13.06 °C, and then declined until 29.20 °C, stabilizing at higher temperatures (Figure 4a). For bio15, the probability increased when precipitation exceeded 18.74 mm, peaked at 160.71 mm, declined until 182.64 mm, and then increased again after 229.16 mm, stabilizing at higher values (Figure 4b). The presence probability for prec2 increased when precipitation was greater than 0 mm, peaked at 82.75 mm, and declined until 286.73 mm (Figure 4c). The probability for bio19 increased with precipitation, peaking at 381.41 mm, declined until 919.44 mm, and then increased again after 3162.45 mm, stabilizing at higher levels (Figure 4d). For prec7, the probability decreased with increasing precipitation, reaching a minimum at 59.39 mm, then increased at 603.63 mm, decreased again at 717.01 mm, and increased once more after 1351.96 mm, stabilizing at optimal levels (Figure 4e). The probability for bio13 increased with precipitation up to 196.46 mm, then fluctuated before rising gradually after 608.44 mm (Figure 4f). Lastly, the probability for bio2 decreased with rising temperatures until 3.78 °C, reached an optimum at 13.32 °C, decreased again until 14.56 °C, and increased after 17.93 °C, stabilizing at higher temperatures (Figure 4g).

**Figure 5:**
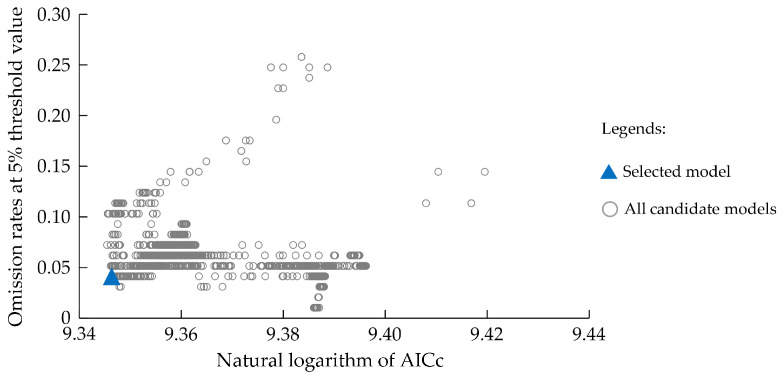
Omission rates and AICc values for all candidate models, with selected models chosen based on statistical significance, omission rates, and AICc criteria.

In the optimized MaxEnt model, the RM value was 1.5 and the FC was LT (Figure 5).

### 3.2. Predicted Environmental Suitability of T. absoluta Under Near-Current Conditions

**Figure 6:**
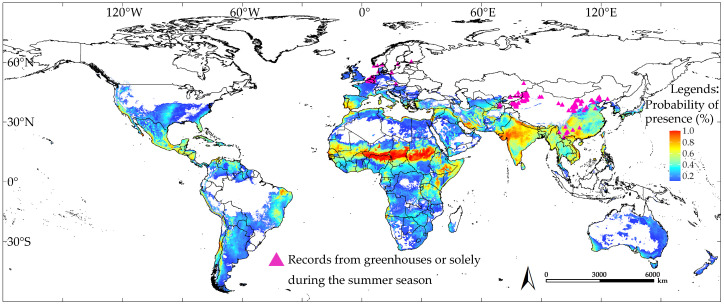
Predicted climatic suitability under near-current climate conditions based on the natural occurrence records of *T. absoluta*; higher values represent greater suitability, while non-shaded areas indicate climatic unsuitability.

**Figure 7:**
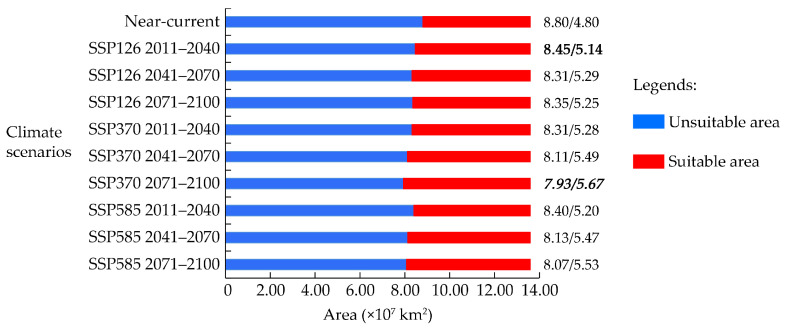
Land areas showing climatic suitability and unsuitability for *T. absoluta* as modeled by MaxEnt, under near-current and future climate conditions. Bold numbers indicate the lowest predicted risk of *T. absoluta* invasion and spread; bold and italicized numbers indicate the highest predicted risk of *T. absoluta* invasion and spread.

The MaxEnt model produced consensus maps depicting suitable and unsuitable areas for *T. absoluta*, as shown in Figure 6. We calculated the areas of climatic suitability and unsuitability for *T. absoluta* under near-current and future climate conditions (Figure 7). Under near-current climate conditions, the potential climatic suitability area for *T. absoluta* was 4.80 × 10^7^ km^2^, accounting for 35.29% of the world’s land area, excluding Antarctica (Figure 6). Our predicted results indicate that a significant portion of the world remains suitable for *T. absoluta*.

### 3.3. Global Climatic Suitability for T. absoluta Modeled with MaxEnt Using the Species’ Natural Occurrence Records Under Future Climate Scenarios

**Figure 8:**
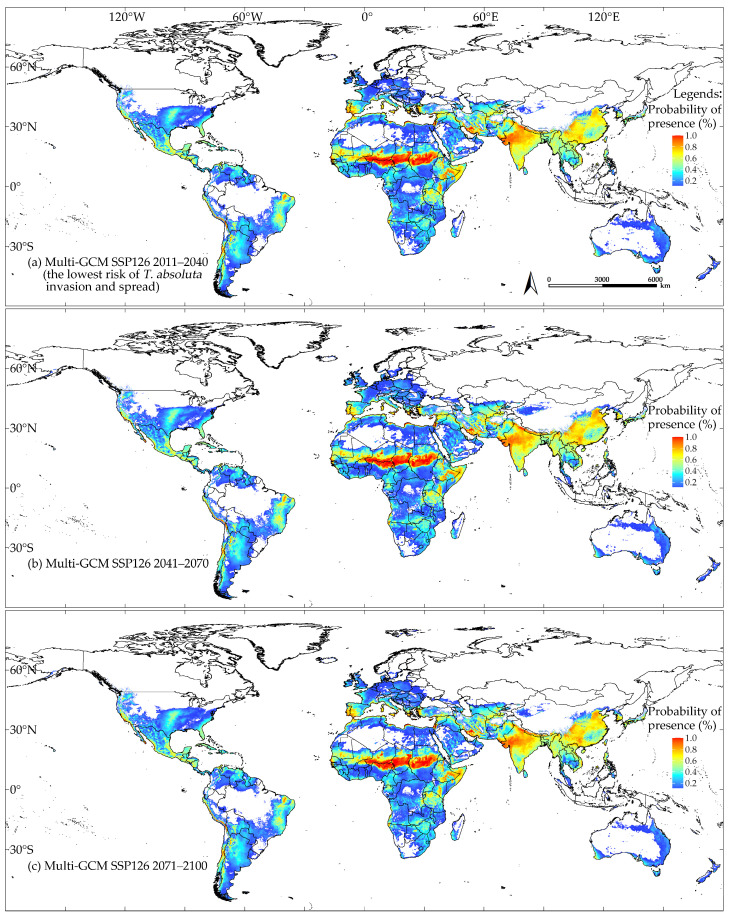
Climatic suitability of *T. absoluta* predicted by the multi-GCM under the SSP126 scenario: proportion of models predicting suitability (%); higher values represent greater suitability, while non-shaded areas indicate climatic unsuitability: 2011–2040 (**a**), 2041–2070 (**b**), and 2071–2100 (**c**).

The model outputs indicated that the multi-GCM anticipated the lowest risk of *T. absoluta* invasion and spread for the period in 2011–2040 under the SSP126 scenario, which assumes low greenhouse gas emissions (Figure 8a). Under this scenario, the areas of climatic suitability decreased by 0.34 × 10^7^ km^2^, approximately 6.08% compared to near-current climate conditions (Figure 7 and Figure 8a).

**Figure 9:**
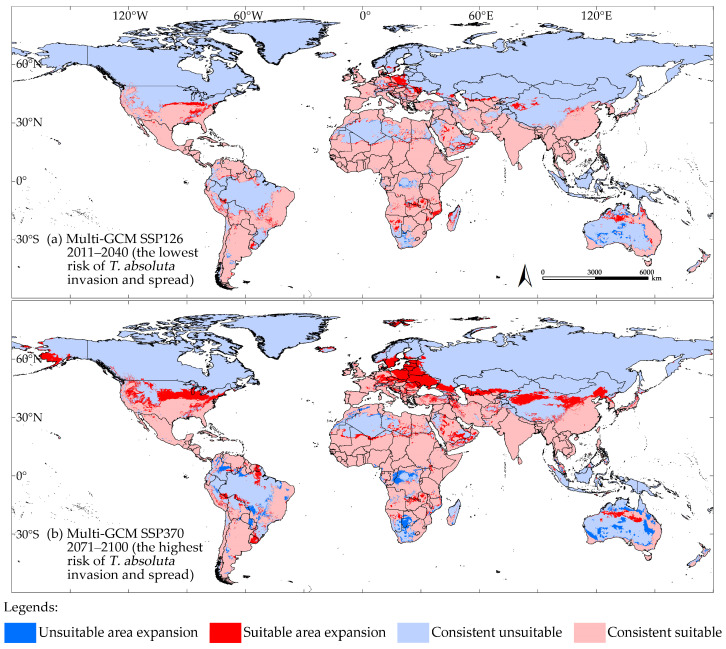
In comparison to the model outputs under near-current climate conditions, the multi-GCM predicted the lowest (**a**) and highest (**b**) probabilities of *T. absoluta* invasion and spread under future climate conditions.

Figure 9a illustrates the shift in climatic suitability for *T. absoluta* under the lowest risk scenario for its invasion and spread. In the Americas and Africa, the potential climatic suitability area for *T. absoluta* expanded, primarily around the periphery of the existing suitable areas. In Europe, the expansion toward higher latitudes is not significant, but a noticeable expansion is observed at the same latitude (around 50° N). In Oceania, particularly in Australia, suitable habitats show a clear expansion at the same latitude (around 20° S). In Asia, a distinct transition from unsuitable to suitable habitats is observed in the northwest of China (around 40° N). The areas of potential climatic suitability and unsuitability for *T. absoluta* that remained unchanged were 4.71 × 10^7^ km^2^ and 8.36 × 10^7^ km^2^, respectively. The areas of potential climatic suitability and unsuitability that increased were 0.43 × 10^7^ km^2^ and 0.09 × 10^7^ km^2^, respectively (Figure 9a).

**Figure 10:**
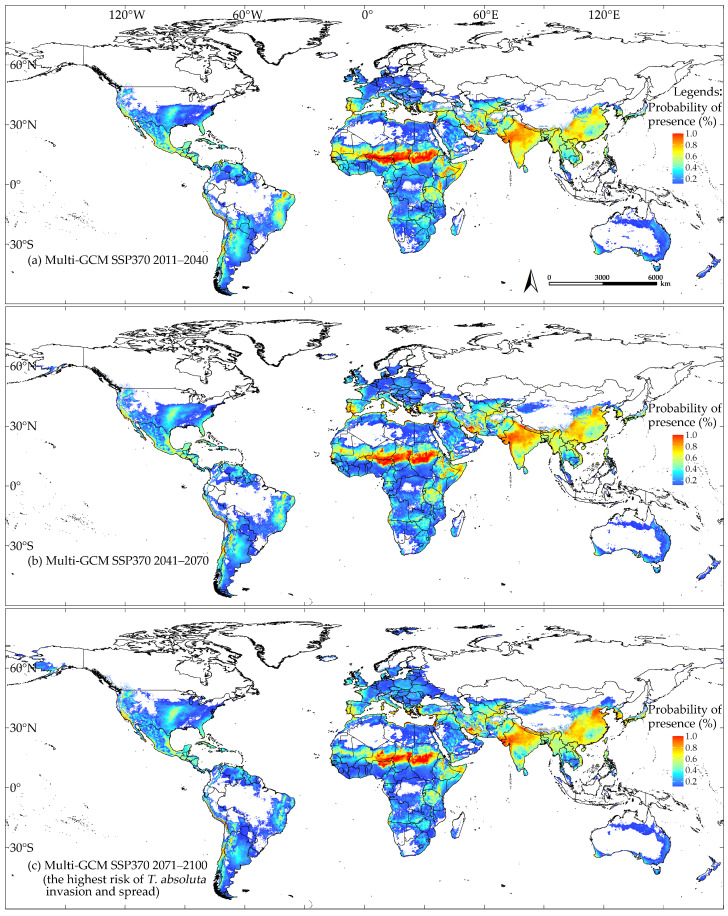
Climatic suitability of *T. absoluta* predicted by the multi-GCM under the SSP370 scenario: proportion of models predicting suitability (%); higher values represent greater suitability, while non-shaded areas indicate climatic unsuitability: 2011–2040 (**a**), 2041–2070 (**b**), and 2071–2100 (**c**).

Conversely, the multi-GCM predicted the highest risk of *T. absoluta* invasion and spread for the period in 2071–2100 under the SSP370 scenario, which assumes high greenhouse gas emissions (Figure 10c). In this scenario, the areas of climatic suitability were approximately 0.87 × 10^7^ km^2^, about 18.13% more than those predicted under near-current climate conditions (Figure 7 and Figure 10c). As shown in Figure 9b, in North America, the potential climatic suitability area for *T. absoluta* primarily expanded between 35° N and 50° N, with a slight increase observed in the southern part of Alaska at higher latitudes. In South America and Africa, the expansion of potential climatic suitability and unsuitability is more scattered and fragmented. In Europe, the expansion is particularly noticeable, concentrated between 45° N and 60° N, and between 10° E and 40° E. In Asia, the expansion of potential climatic suitability is mainly concentrated between 25° N and 45° N, moving toward higher latitudes. In Oceania, the expansion of potential climatic unsuitability in Australia is much more significant than that of suitability. The areas of potential climatic suitability and unsuitability for *T. absoluta* that remained unchanged were 4.51 × 10⁷ km^2^ and 7.64 × 10⁷ km^2^, respectively. The areas of potential climatic suitability and unsuitability that increased were 1.16 × 10⁷ km^2^ and 0.30 × 10⁷ km^2^, respectively (Figure 9b). The model output from multi-GCM under the SSP585 scenario is shown in Figure S1.

## 4. Discussion

### 4.1. Limitations and Uncertainties

To investigate the relationship between species and the environment and to develop environmental suitability predictions, it was necessary to filter the environmental variables before modeling [76]. Elevation was selected as one of the environmental variables in our analysis of species’ environmental suitability, in line with the methodology proposed by Liu et al. and Yang et al. [12,22]. The pool of candidate variables in this study included elevation and 67 climate variables. After determining that recent climate change has not significantly affected the species’ distribution over the past 44 years, in 1981–2024, and excluding records from greenhouses and summer-only occurrences, elevation was not selected for inclusion in the final model. In datasets with limited sampling, the influence of elevation is often exaggerated, while the role of climate variables is underestimated [77]. Additionally, the negative impact of elevation on the model’s transferability highlights concerns about its effectiveness in evaluating pest invasion risks [78]. This result indicates that the dataset used for modeling is robust and comprehensive, with no significant under-sampling issues overall. However, localized under-sampling is evident in the native range of *T. absoluta*, particularly in South American countries, such as Venezuela, Colombia, Ecuador, Bolivia, Paraguay, and Uruguay, where no new records of the species’ natural occurrences have been recorded in 2011–2024. In contrast, central Chile—likely the primary source region for the initial invasion of *T. absoluta* into Europe—shows high climatic suitability under the current conditions, with a predicted presence probability approaching 1.0. This finding is consistent with the known invasion history of the species [1,3].

The Human Influence Index, among other factors, significantly influences the pest risk analyses of crop insect pests, potentially having the greatest impact [79,80]. However, future projections are significantly influenced by individual decisions made by farmers and market developments, making them challenging to model [20]. Therefore, we chose to focus solely on the non-human factors’ effects on the environmental suitability of *T. absoluta*.

Temperature is the most crucial environmental factor influencing *T. absoluta*, with both low and high temperatures impacting its survival rate [81]. It was found that the survival of *T. absoluta* was not significantly impacted at 0 °C during its developmental stage [82]. When exposed for 6, 8, and 10 h, the 50% lethal temperature for *T. absoluta* larvae remained stable, ranging from −3.29 to −3.53 °C [17]. This range is slightly lower than the suitable temperature of −2.81 °C predicted by the tas2 response curves generated by the MaxEnt model, with a difference of 0.48 °C. However, this discrepancy is within a reasonable range, as our model incorporates the climatic data from multiple geographic populations. Given the broader geographical scope and the inherent variability of temperature thresholds across regions, some variation between model predictions and localized biological studies is expected [83]. The close alignment between our model outputs and the biological findings further demonstrates the robustness of the model, reinforcing its ability to predict lethal temperature thresholds under varying climatic conditions.

### 4.2. Model Evaluation

Upon reviewing various ecological niche models for *T. absoluta*, we observed differences in the conclusions across different periods. Tonnang et al. indicated that *T. absoluta* had a high probability of long-term survival in regions including North America, South America, Central and Southern Africa, areas surrounding the Mediterranean, and most parts of Southeast Asia, where the ecoclimatic index (*EI*) exceeded 50 [18]. Compared to our predictions, these results overestimate the environmental suitability in *T. absoluta*’s native range in South America and underestimate it in Central Asia [18]. Their dataset contains a higher proportion of natural occurrence records from *T. absoluta*’s native range. A significant disparity was observed between the potential niche estimated using occurrence records from invasive ranges and those from the native range [32]. Despite the consistent predictive performance for both the native-range and invasive-range models, the areas identified as highly environmentally suitable differed [84].

Santana et al. used occurrence records in 1960–2017 and the CLIMEX model to predict the global potential distribution of *T. absoluta* [10]. Under near-current climate conditions, the climatic suitability of *T. absoluta* from our model output closely matched Santana et al.’s findings. Similar to our approach, both Early et al. and Santana et al. excluded greenhouse and summer-only occurrences from their data selection [10,13]. However, Santana et al. overestimated the environmental suitability for *T. absoluta* invasion and spread in Europe north of 60° N under future climate conditions, while underestimating the environmental suitability in large areas of Africa, Asia, and the native region in South America [10]. The lack of essential information regarding the environmental niche of species can significantly impact the model’s outcomes, as the climatic niche is a subset of the potential niche in which the species actually persists [54]. While modeling methods can address disparities between the climatic and potential niches, missing essential niche information hinders the accurate representation of *T. absoluta*’s environmental niche. We confirmed that climate change has a minimal impact on established species’ areas and then utilized an expanding-niche dataset of *T. absoluta* that incorporates more essential ecological niches for modeling.

Fand et al. predicted that suitable areas for *T. absoluta* in India were primarily located in southwest India and were smaller compared to the model outputs [21]. In our study, we found that nearly all areas in India, except for the northwest corner, were suitable for *T. absoluta*. Xian et al. used the CLIMEX model to predict the potential distribution of *T. absoluta* in China [19]. Their model indicated that almost all areas east of 105° E in China were moderate to high-risk areas (*EI* = 20–60) for *T. absoluta*. In contrast, our model outputs revealed that the regions north of 35° N and east of 105° E in China were climatically unsuitable for *T. absoluta*. Yang et al. used the MaxEnt model to predict that western China is a suitable area for *T. absoluta* [22]. However, our findings suggest that, in most of the northern and central regions of Xinjiang, *T. absoluta* may not support overwintering, while southern Xinjiang shows an increased potential for overwintering [17]. Significant climatic suitability changes in central Xinjiang were predicted under future climate conditions. Furthermore, Yang et al.’s study did not exclude the occurrence records of species found in greenhouses or solely during the summer season, which could lead to an overestimation of the species’ environmental suitability. Jinyu et al. used the CLIMEX and MaxEnt models to predict the potential distribution of *T. absoluta* in China [15]. They retained species occurrence points north of 50° N in Europe and near 45° N in northwest China for model prediction. In contrast, our study excluded these points during modeling. Furthermore, compared to their results, our model predictions indicate that *T. absoluta* exhibits potential climatic suitability in eastern China, particularly between 34° N and 40° N.

### 4.3. Dispersal and Invasion Control

In the absence of complex terrain barriers, climate change-induced warming is expected to facilitate the continuous migration of species and entire biotic communities toward higher latitudes or altitudes [81]. These shifts are anticipated to occur more rapidly in high-latitude regions compared to those closer to the equator, due to the anticipated faster warming in these areas [85,86]. According to the multi-GCM predictions, under the highest risk scenario of *T. absoluta* invasion and spread (SSP370, assuming high greenhouse gas emissions), climatic suitability is projected to increase predominantly in areas at higher latitudes or altitudes. In contrast, under the lowest risk scenario (SSP126, assuming low greenhouse gas emissions), the increase in climatic suitability is less pronounced. This suggests that, as greenhouse gas emissions rise, *T. absoluta* is likely to invade more regions. In areas where farmers lack preventive measures, this could lead to more frequent biological invasions and associated economic losses.

The actual invaded range of *T. absoluta* may far surpass the potential distribution predicted by ENMs. *Tuta absoluta* typically overwinters beneath surface litter or in the soil [87]. When there is a sudden drop in temperature, farmers have observed that *T. absoluta* flies into heated greenhouses or homes, where it can survive the winter [88]. Due to its broad host range and high adaptability, *T. absoluta* does not enter diapause, even when it goes without feeding for extended periods [89]. Through long-term colonization in cold regions with high latitudes or altitudes, *T. absoluta* may gradually adapt to colder environments and expand its damage range [90]. The pest exhibits a high reproductive capacity; even as few as 10% of surviving individuals can cause substantial damage in the following growing season [82].

China is the world’s largest producer of tomatoes. In 2021, the country’s tomato cultivation area reached 1.113 million hectares, accounting for 22.02% of the global tomato-growing area, with greenhouse cultivation covering 57.2% of the total tomato-growing area [91]. In China, *T. absoluta* has spread to more than 12 provinces and is frequently reported feeding on tomatoes in greenhouses and other horticultural facilities [92]. Greenhouses infested with *T. absoluta* often serve as breeding grounds for further invasion and expansion [3]. The extensive use of greenhouses has facilitated the rapid spread of the pest into environmentally unsuitable areas, at a rate of approximately 600 km per year in northern China.

In southwestern and central China, the climatic suitability for *T. absoluta* is high, and these regions are well-suited for open-field tomato cultivation. However, due to the significant yield advantage of greenhouse cultivation over open-field production, there is also a large area of facility-grown tomatoes. Despite this, open-field tomato production shows a positive carbon ecological efficiency, while facility-grown tomato production carries a negative environmental externality [91]. Additionally, the presence of these artificial structures increases the difficulty of controlling *T. absoluta*.

Based on the global occurrence records of *T. absoluta* and the potential climatic suitability predicted by the MaxEnt model, this study provides the following control recommendations: (1) In areas with high climatic suitability for *T. absoluta*, strengthen quarantine measures to detect the early colonization of the pest. (2) In areas where *T. absoluta* is not climatically suitable, continuous exposure to cold shocks during winter can significantly increase mortality [17], helping to control pest populations. (3) In regions suitable for open-field tomato cultivation, reduce greenhouse use where possible to decrease the difficulty of controlling *T. absoluta*, thereby reducing the cost of chemical control and minimizing ecological damage, achieving a balance between economic profit, ecological benefit, and pest control efficiency.

## 5. Conclusions

In this study, we utilized an optimized MaxEnt model based on datasets representing expanding-niche spaces. The multi-GCM predicted the lowest risk of *T. absoluta* invasion and spread for the period in 2011–2040 under the SSP126 scenario, whereas the highest risk was predicted under the SSP370 scenario for the period in 2071–2100. The primary factor influencing *T. absoluta*’s climatic suitability was identified as the mean daily air temperature in February (tas2). Greenhouses, in particular, provide viable pathways for *T. absoluta*’s invasion and spread in regions where it cannot naturally overwinter. We strongly advocate for enhanced quarantine measures in greenhouses and other artificial facilities, especially in regions predicted to be unsuitable by ENMs, to effectively mitigate potential invasions. Our findings provide valuable insights for researchers aiming to improve ENMs, emphasizing the importance of using datasets that include more essential environmental niche information after considering the impact of climate change on species distribution to enhance predictive accuracy. This comprehensive assessment of *T. absoluta*’s global climatic suitability offers crucial guidance for developing strategies to prevent its spread in global tomato-growing regions.

## Figures and Tables

**Table 1:** Global climate models used to make future projections in this study.

GCM	Code	Priority	ECS
UKESM1-0-LL	UK	2	5.4
IPSL-CM6A-LR	IP	4	4.6
MRI-ESM2-0	MR	5	3.1
MPI-ESM1-2-HR	MP	3	3.0
GFDL-ESM4	GF	1	2.7

**Table 2:** Evaluation of MaxEnt model overfitting and predictive accuracy using test dataset metrics.

Overfitting Test		Predictive Accuracy						
OR_mtp_	OR_10_	AUC	AUC ratio	TSS	Kappa	Boyce	Jaccard	Sørensen
0.005	0.141	0.907	1.520	0.959	0.815	0.983	0.829	0.906

**Table 3:** Climatic variables selected for the modeling process.

Code	Percent Contribution	VIF
tas2	57.0	1.09
bio15	15.3	1.13
prec2	10.1	1.34
bio19	7.3	1.26
prec7	0.6	1.60
bio13	5.8	2.04
bio2	4.0	1.18

## Data Availability

The global gridded climate data and climate projections are available from Climatologies at High resolution for the Earth’s Land Surface Areas (CHELSA, available online: chelsa-climate.org, accessed on 1 June 2024). Distribution records of *Tuta absoluta* are available from three online databases: (1) Global Biodiversity Information Facility (GBIF, available online: www.gbif.org, accessed on 8 October 2024), (2) European and Mediterranean Plant Protection Organization (EPPO, available online: https://gd.eppo.int/, accessed on 8 October 2024), and (3) Centre for Agriculture and Bioscience International (CABI, available online: www.cabi.org, accessed on 8 October 2024), as well as from the published studies (denoted in the main text).

## Supplementary Materials

The following supporting information can be downloaded at: https://www.mdpi.com/article/10.3390/insects16020185/s1, Table S1: Environmental variables used for predicting the environmental suitability of *Tuta absoluta*. Figure S1: Climatic suitability of *T. absoluta* predicted by the multi-GCM under the SSP585 scenario.
