# Supplementary material for: Modeling the Potential Climatic Suitability and Expansion Risk of Tuta absoluta (Meyrick, 1917) (Lepidoptera: Gelechiidae) Under Future Climate Scenarios
Source: Insects. 2025 Feb 9;16(2):185. doi: 10.3390/insects16020185 (PMC11856755; doi:10.3390/insects16020185)
Supplement: Supplementary file 1 [file insects-16-00185-s001.zip › insects-3444165-supplementary.pdf]

Table S1. Environmental variables used for predicting the environmental suitability of *Tuta absoluta*.

Environmental variable	Code
Mean annual air temperature	bio1
Mean diurnal air temperature range	bio2
Isothermality	bio3
Temperature seasonality	bio4
Mean daily maximum air temperature of the warmest month	bio5
Mean daily minimum air temperature of the coldest month	bio6
Annual range of air temperature	bio7
Mean daily mean air temperatures of the wettest quarter	bio8
Mean daily mean air temperatures of the driest quarter	bio9
Mean daily mean air temperatures of the warmest quarter	bio10
Mean daily mean air temperatures of the coldest quarter	bio11
Annual precipitation amount	bio12
Precipitation amount of the wettest month	bio13
Precipitation amount of the driest month	bio14
Precipitation seasonality	bio15
Mean monthly precipitation amount of the wettest quarter	bio16
Mean monthly precipitation amount of the driest quarter	bio17
Mean monthly precipitation amount of the warmest quarter	bio18
Mean monthly precipitation amount of the coldest quarter	bio19
Mean daily maximum air temperature	tasmax1–12
Mean daily air temperature	tas1–12
Mean daily minimum air temperature	tasmin1–12
Monthly precipitation amount	prec1–12
Elevation	elev

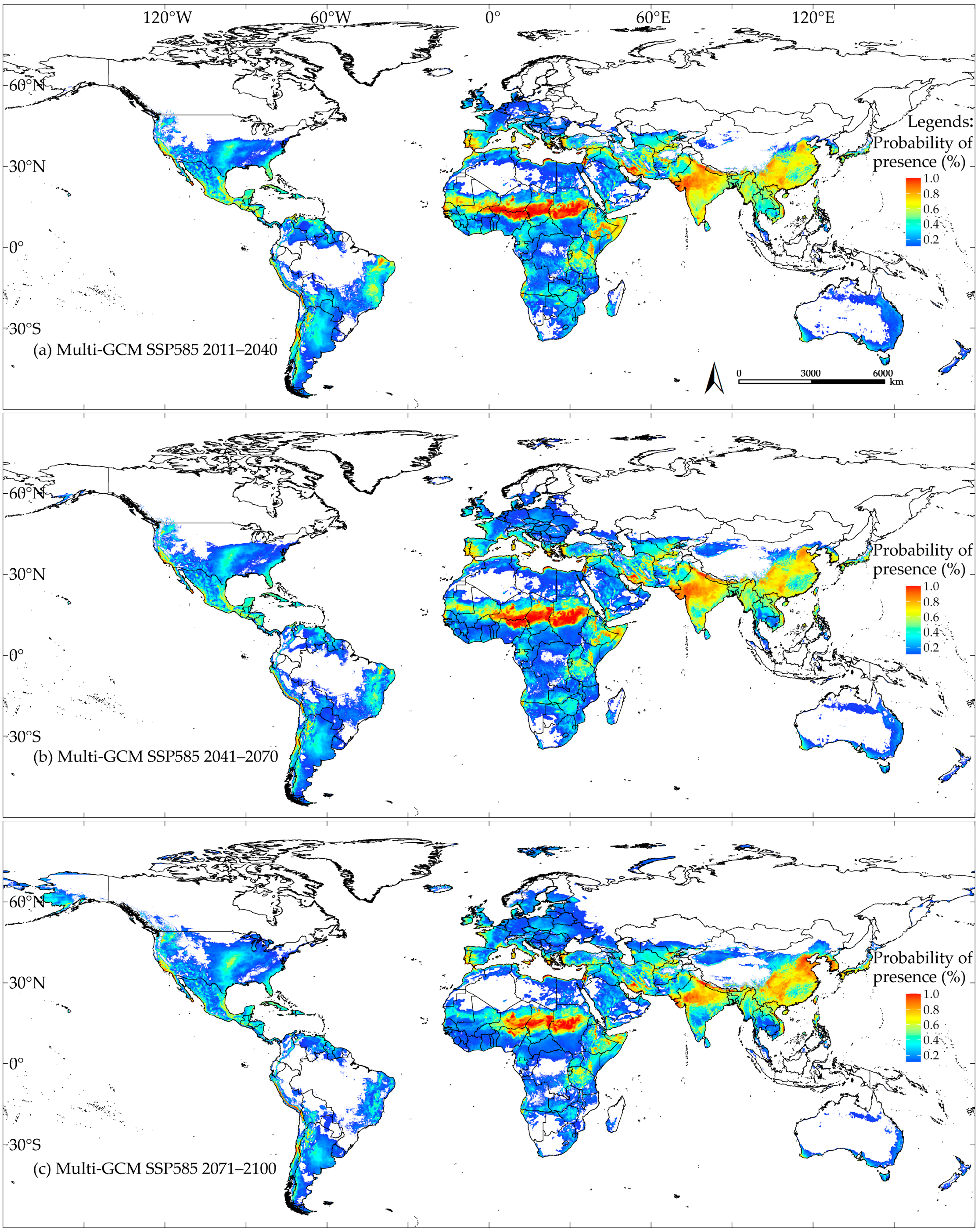

Figure S1. Climatic suitability of *T. absoluta* predicted by the multi-GCM under the SSP585 scenario: proportion of models predicting suitability (%); higher values represent greater suitability, while non-shaded areas indicate climatic unsuitability. 2011–2040 (**a**), 2041–2070 (**b**), and 2071–2100 (**c**).
